# Spatial Ecology of Estuarine Crocodile (*Crocodylus porosus*) Nesting in a Fragmented Landscape

**DOI:** 10.3390/s16091527

**Published:** 2016-09-19

**Authors:** Luke J. Evans, T. Hefin Jones, Keeyen Pang, Silvester Saimin, Benoit Goossens

**Affiliations:** 1 Cardiff School of Biosciences, Cardiff University, Cardiff CF10 3AX, UK; jonesth@cardiff.ac.uk; 2 Danau Girang Field Centre, c/o Sabah Wildlife Department, Wisma Muis, 5th Floor, Block B, Kota Kinabalu 88100, Malaysia; 3 Sabah Wildlife Department, Wisma Muis, 5th Floor, Block B, Kota Kinabalu 88100, Malaysia; silvester.saimin@sabah.gov.my; 4 Sustainable Places Research Institute, Cardiff University, 33 Park Place, Cardiff CF10 3BA, UK; 5 Hornbill Surveys Sdn Bhd, Lot 9, Harapan Baru Light Ind Estate, Mile 8, Jalan Labuk, Sandakan 90009, Malaysia; intrajasa@gmail.com

**Keywords:** drone, UAV, salt water, aerial survey, Borneo, Sabah, reptile

## Abstract

The role that oil palm plays in the Lower Kinabatangan region of Eastern Sabah is of considerable scientific and conservation interest, providing a model habitat for many tropical regions as they become increasingly fragmented. Crocodilians, as apex predators, widely distributed throughout the tropics, are ideal indicator species for ecosystem health. Drones (or unmanned aerial vehicles (UAVs)) were used to identify crocodile nests in a fragmented landscape. Flights were targeted through the use of fuzzy overlay models and nests located primarily in areas indicated as suitable habitat. Nests displayed a number of similarities in terms of habitat characteristics allowing for refined modelling of survey locations. As well as being more cost-effective compared to traditional methods of nesting survey, the use of drones also enabled a larger survey area to be completed albeit with a limited number of flights. The study provides a methodology for targeted nest surveying, as well as a low-cost repeatable flight methodology. This approach has potential for widespread applicability across a range of species and for a variety of study designs.

## 1. Introduction

The history of crocodilian nesting studies began in the 1960s [1,2], although detailed comparative work on the nesting behaviour of various species did not commence until a decade later. *Crocodylus porosus* nesting was first examined in detail by Webb et al. [3]. This early work focussed on the mechanics of nesting behaviour (for example, what building materials were used), and recording nest characteristics such as temperature and the number of eggs oviposited. Spatial analyses, or assessments of the distribution of crocodile nests in any given area, have not been the focus of many studies. Possibly the best example, to date, is work carried out by Harvey and Hill [4]. Using Landsat™ image analysis (see below) and aerial photography to classify areas by vegetation types, they used a Boolean overlay approach to determine potentially suitable breeding habitat [5]. During this study, aerial photography was found to be far more useful in the classification of nesting habitats [4].

There have been a couple of key factors that have limited the effectiveness of nesting studies, particularly when attempting to calculate nest density in a particular area. Firstly, it is very difficult to determine that all nests in a region have been discovered; thus, nest density calculations are always rough approximations and usually underestimates. Secondly, studies of this nature are often prohibitively expensive, as they rely on the use of helicopters and airboats, or time-costly walked surveys. The use of modern/advanced technology to examine crocodilian nesting behavior is a new development and this study builds on a drone-based methodological paper, which examined the potential use of novel drone technology in finding crocodile nests [6].

The use of drone (or unmanned aerial vehicle (UAV)) technology in conservation and biological management programmes is a relatively recent development [7,8]. Over the past decade, drones have been employed to service a host of ecological needs. Applications such as the monitoring of Eurasian beaver (*Castor fiber*) reintroductions [9], surveying tree falls [10], identifying of forest gaps [11] and nesting of canopy birds [12] are examples of research and monitoring fields within which the technology has been applied. Drones are frequently used in active conservation practice; for example, in the monitoring of poaching activities perpetrated against both the black (*Diceros bicornis*) and white (*Ceratotherium simum*) rhinoceroses [13]. The flexibility of the technology to perform in remote and urban locations alike means that, as long as weather conditions allow, drones can be a financially accessible method of surveying most areas on Earth.

One challenge encountered when using traditional aerial photography is that some areas are obscured from view; this is particularly problematic in forested areas. The use of drones in the determination of crocodile nesting distribution could demonstrate the feasibility and applicability of the technology to other facets of crocodilian research. Martin et al. [14], for example, have already demonstrated that adult alligators could be easily identified aerially using drone technology, suggesting that count surveys and future population density estimates could, at least in part, be calculated in this way.

Selective land conversion for agricultural purposes raises a number of potential issues for crocodile nesting. Prevalent throughout tropical regions, oil palm (*Elaeis guineensis*) plantations are expanding rapidly into previously remote areas [15,16]. The implications of this for estuarine crocodile (*C. porosus*) nesting are unknown; crocodiles are frequently seen in plantation areas (*pers. obs*.). Preliminary work carried out by Evans et al. [6] indicated that crocodiles will nest in areas of medium to high anthropogenic disturbance, and within close proximity to oil palm plantation. This could be indicative of either insufficient nesting habitat being available or individuals continuing to utilise successful nest sites even after their surrounding environment has been altered. There are numerous other less-well examined effects that could influence both the likelihood of successful nesting, as well as post-hatching survival of the estuarine crocodile. Oil palm plantations require a non-natural, irrigation system to ensure sufficient water for crop development; they are, however, unable to withstand long periods of flooding [17]. These artificial hydrological systems are unlikely to benefit hatchling dispersal due to the lack of connectivity between irrigation ditches, and are also likely to bring young crocodiles into closer proximity to potential predators such as monitor lizards [18].

Utilisation of drone surveys enables high-resolution identification of crocodile nests; this allows more accurate mapping of their spatial distribution [6]. This present study aimed to identify all possible estuarine crocodile nests within a specific region of the Kinabatangan River in Sabah, Malaysia. The study site is known to harbor a resilient population of estuarine crocodiles, compared to other sections of the Kinabatangan River, as well as to neighboring rivers in Sabah [19]. It was hoped that baseline data for crocodile nesting in a tropical freshwater ecosystem could be established. Nest sites can, as previously discussed, be found in semi-predictable locations owing to their proximity to permanent water sources, as well as their prevalence in certain types of habitat [4,20,21]. Given that much of *C. porosus* habitat is comprised of closed canopy, and the aforementioned predilection for swampland, particular targeting of ‘suitable’ nesting habitat was possible. In an effort to validate the applicability of the predictive modeling, additional areas were surveyed within the study site.

Five hypotheses were tested. Firstly, nest site density is higher in exposed, open-canopy areas than under dense forest cover with walked “closed-canopy” recces (Hypothesis 1). Female nest site selection can be predicted in terms of habitat and can therefore be selectively surveyed through the use of predictive modelling and aerial drone technology (Hypothesis 2). Nest sites are solitary, with individuals actively choosing nest sites that are spatially independent of other nesting females (Hypothesis 3). Estuarine crocodiles select nest sites in a non-random fashion given habitat availability constraints (Hypothesis 4), thus validating the use of drones as a tool for nesting surveys. Finally, building on findings from Evans et al. [6], nesting can occur in the presence of medium to high levels of human disturbance, implying that the presence of oil palm plantations is not necessarily a barrier to successful nesting (Hypothesis 5).

## 2. Methods

The study was carried out over a 35 km stretch of the Kinabatangan River. The stretch was selected due to the presence of high numbers of both adult and juvenile crocodile individuals (unpublished data). The second longest river in Borneo, and, at 560 km, the longest in the eastern Malaysian state of Sabah, the Kinabatangan River has a catchment of around 16,800 km^2^, an area encompassing around 23% of the total land area of Sabah [22,23]. Within this catchment area lies the Lower Kinabatangan Wildlife Sanctuary (LKWS), consisting of 10 distinct forest “lots” covering an area of 27,960 ha [15]. The landscape consists of a highly fragmented forest-oil palm matrix, with forested areas being largely degraded secondary forest. This type of forest results in patchy areas of closed canopy forest interspersed with open grassland and areas with very sparse partial tree coverage. The Kinabatangan region supports a large population of estuarine crocodiles (*C. porosus*); the population has undergone rapid recovery following state-wide protection of the species initiated in 1982 [19].

### 2.1. Walked Recces

Walked recces were initially carried out throughout the 35 km study region to determine whether crocodiles were likely to be utilising areas of closed canopy cover for nesting; the resultant lack of positive nest identification justified the use of aerial drones. These recces were carried out from September–November 2013. These detailed walked recces were conducted over an area totaling 120 km of riparian riverbank habitat, oxbow lake (consisting of a mixture of riparian and seasonally flooded habitats) and swampland habitats (areas where water is present year round). A total of 101 km (84.2%) covered during recces was covered under closed canopy. Each recce was conducted by two observers, each of whom walked parallel to the water source at a distance of 5 m from the water, whilst maintaining 15 m between them, creating total coverage of approximately 20 m.

Of the 120 km of walked recces conducted, 50.8% of the habitat surveyed was riparian riverbank, 27.1% oxbow lake shoreline and 22.1% swampland, across 14 separate nesting transects. These transects (4.5–10 km) were searched, in detail, for any indication of crocodile nesting or presence; for example, footprints or slide marks caused by the dragging of the body through mud, sand or vegetation. Marks found were carefully examined to distinguish them from other animals such as monitor lizards, snakes, bearded pigs or any other ground-dwelling animals found in the region. Transect locations were chosen as being potentially suitable for nesting primarily based on their proximity to permanent water sources [3,4,21]. Each transect was selected to incorporate the highest percentage of closed canopy possible; this allowed determination of whether regions with open access to direct sunlight were more likely to be selected for nesting (Hypothesis 4). The walking of large tracts of the landscape also provided an indication of the different habitats present within the region, and which areas would be most suitable for aerial analysis.

### 2.2. Drone Surveys

Aerial surveys were conducted on the basis that nesting under closed canopy was occurring at such low occurrence that not observing them would have no meaningful impact on estimated nest densities (based on lack of detection during walked surveys). Aerial surveys were conducted with the use of two different drone systems (during 2013 and 2014, respectively). Surveys were carried out during September and October in both survey years. These months were chosen due to observance of hatchling emergence during November and December (*pers. obs*.).

In 2013, an exploratory series of surveys were carried out using a Bormatec Maja™ drone (see Evans et al. [6] for a detailed description of it specifications). As this initial attempt proved successful, a second, more expansive, series of surveys was conducted in 2014, this time utilising the Skywalker™ drone. This equipment provides a more stable and efficient flight. Both drone systems were fixed wing aircraft, able to provide a high degree of stability, whilst ensuring desirable range capabilities. Under typical weather conditions (low wind and no rain), the Skywalker™ was able to conduct flights totaling one hour, whilst carrying a payload of up to 1 kg. In real terms, this equates to flight distances of up to 35 km and search grids of around 550 ha. Two cameras were used for surveys conducted in 2013 and 2014. However, these were of the same make and model (Model S100, Canon, Ota, Tokyo, Japan). A CHDK (Canon Hack Development Kit) script was uploaded to the camera to ensure the camera could take pictures every three seconds without manual triggering.

Other than during take-off or landing, or during emergencies, drone flights were conducted using an Auto Pilot Module (APM). Flights were conducted as close to the planned flight grid as possible (for flight grid description see Evans et al. [6]), whilst allowing for an open landing and take-off site. These “ground stations” were located in a variety of habitats, including riverbanks, grasslands and oil palm plantations. Ensuring that ground stations were located close to search grids allowed for larger areas to be covered within any particular grid, as battery power was not wasted traveling to and from the study site. Post-hoc analysis of aerial photographs taken involved stitching following the methodology explained in Evans et al. [6].

**Figure 1:**
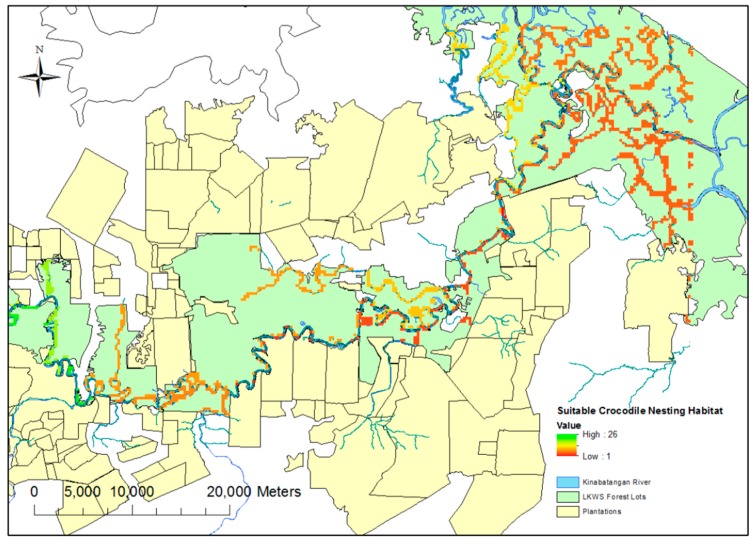
Nesting suitability model for the LKWS. Defined using a “fuzzy overlay” function in ArcGIS. Areas of suitability are defined by the presence of a coloured pixel with increasing suitability defined on a red (low) to green (high) scale. Suitable nesting locations are largely confined to major waterways.

The selection of drone mapping grids was based on ensuring an array of riverine, swamp and oxbow habitat, as well as covering all the major tributaries across the study site. A predictive theoretical model of suitable nesting sites was produced to aid in the selection of these sites (Figure 1). This model was produced using the “fuzzy membership” and “fuzzy overlay” functions in ArcGIS 10; these functions allow the designation of certain required spatial prerequisites (such as distance to permanent water sources) for nesting, whilst discounting other areas based on presumed undesirable geographic traits (for example, proximity to oil palm plantations (distances tailored based on findings from Evans et al. [6]). These traits were derived from the existing literature [4]. Whilst flights were flown to include these “suitable zones”, flights were also conducted outside of these areas to test the efficacy of the predictive modeling.

Once identified, nests sites were ground-validated, where possible, and general ground habitat assessed. Both validated and non-validated nests were subsequently assessed for a series of geographic traits, such as distance to permanent water, distance to canopy and distance to plantation. These descriptive statistics were stated throughout as ±1SE. These variables were used to create a binomial generalised linear mixed model (GLMM) using R (3.1.3) (Table 1). The “lme4” package [24] was used to determine which factors were of greatest importance in determining a nesting location of estuarine crocodiles. The model was refined through the use of a “dredge” model-comparison function carried out with the use of the package “MuMIn” [25]. “Dredging” creates a series of models with subsets of variables in order to find the most reliable model. Conditional and marginal R^2^ values were then used to assess the level of variance explained by both fixed and random model terms. Finally, model predictions were made to evaluate the role proximity to plantation plays in nesting location choices.

## 3. Results

Walked recces in closed canopy failed to identify any nests, providing evidence that the majority of nesting occurs in areas of open, canopy-devoid, areas. During the 2013 field season, a total of 1550 aerial ha were surveyed using drones and three potential nests identified. Two of these were confirmed as true nesting locations. A further 5160 ha were surveyed during 2014; this resulted in a total area surveyed over two field seasons of 6710 ha.

**Figure 2:**
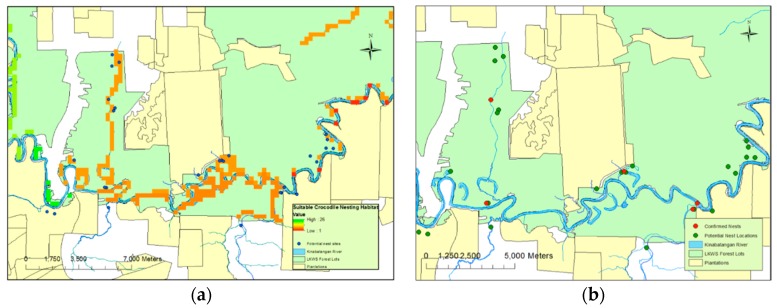
(**a**) Potential nest sites in relation to habitat suitability model; the majority of nests sites fell inside of, or close to, identified suitable areas within the study site. Suitability defined as areas of coloured pixels as in Figure 1, with potential nest sites overlaid as blue dots; (**b**) Locations of confirmed nest sites showing close proximity to water, as well as, on three occasions, close proximity to oil palm plantations.

Twenty six potential nests were identified during 2014; however, flooding of a large part of the study site during the field season prevented verification of 10 of these nests. Those nests that were flooded were not verified for either safety or lack of accessibility reasons. Nine of these unverified nests were excluded from analysis in order to retain the rigor of the model. One flooded nest was included despite the lack of ground verification based on similarities between its aerial image and those of previously ground-verified nests. Of the drone-facilitated nesting surveys carried out over two field seasons, 2013 and 2014, a total of 29 potential nests were identified. Of these, four were confirmed as actual crocodile nests with the addition of one unverified nest (two in 2013 and two in 2014) (Figure 2a). A total of 15 nests were highlighted as potential nests from the aerial photography that were later found to consist of accumulations of dead material unassociated with crocodilian nesting behaviour.

### 3.1. Habitat Suitability

Of the five nests, all were located in close proximity (mean 13.9 ± 12.9 m) to permanent water sources. They were found within small open areas and within close proximity (22.2 ± 14.3 m) of closed canopy cover (Figure 2b). While plantations were, generally, not included in the surveyed areas, one nest was found close to a plantation border; across all nests identified, the nests were a distance of 374 m (±139.7) from such boundaries. Four of the confirmed nests were located within the protected habitat lots of LKWS. One nest was located outside, in privately-owned land that could be open to conversion. Neither nest site located during 2013 was reutilised during 2014, and all nests were spatially independent (Figure 2b). Three of the five nests were located in “drying” or “old” oxbow lakes; each of these had a permanent aquatic connection to a main water body, such as a large tributary. The other two nests’ locations, including the unverified nest, were directly adjacent to a major water body.

Nest sites could not be attributed to specific females and no instances were recorded of females guarding their nests. There was, however, evidence of females spending time at the nest site, and of excavation of eggs during hatching. Wallows (depressions containing mud or shallow water) were recorded around one of the nest sites. One nest located during 2013 surveys was visited the day after hatching and visual confirmation of 19 hatchlings was recorded. Egg membranes and shells were collected, and evidence of at least 24 successful hatchings was found. There was no evidence of pre- or post-hatchling mortality.

### 3.2. General Linear Mixed Model

**Figure 3:**
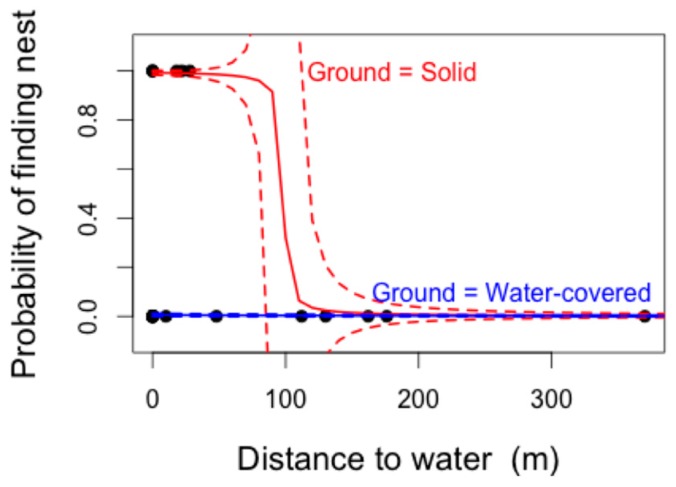
Plotting predictions from binomial GLMM. Model provides a binomial predictive distribution, indicating that nesting is less likely further away from permanent water sources. Solid lines denote predicted probability; with dashed lines showing the error associated with the probability levels. Data included both confirmed nest sites as well as those that were “potential” and later discounted nest sites. Despite trajectory of confidence intervals, prediction could not be less than zero.

The most parsimonious GLMM model structure to explain the presence/absence of crocodile nests (lowest AIC–Akaike Information Criterion–identified using the “dredge” function) included the variables “distance to water” and “ground water presence”. This model also yielded the greatest model weight (W = 0.195), and was therefore considered the best model structure to explain the model variables included. Distance to water was significantly negatively correlated with the likelihood of finding a nesting site (F_1,29_ = 5.59, *p* = 0.018). The presence of ground water was close to significance (F_3,29_ = 7.36, *p* = 0.061), with the presence of less than 1 m of standing ground water resulting in a higher likelihood of nesting (Figure 3). Marginal (R^2^m) and conditional (R^2^c) r-squared values showed that the majority of the variance being described by the model was derived from the fixed terms (distance to water and standing ground water), with negligible variation explained by the random term, year (R^2^m = 0.571, R^2^c = 0.571).

Predictions based on this model suggested that whilst locations of nests in standing water could not be easily predicted, nests on solid ground were very likely to be less than 100 m away from permanent water sources (Figure 2b). In reality, these predictions are in line with those used during the original “fuzzy overlay” modelling (Figure 2a). An increase in sample size could lead to a refinement of solid-ground predictions and lead to more stringent standing water predictive sampling.

There was a marked difference in image quality between the 2013 (higher quality) and 2014 (lower quality) drone surveys, despite the use of two cameras of the same make and model. This could have been a result of different light conditions (see Evans et al. [16]), or the speed of travel of the drone. Issues with image clarity and stitching quality were largely linked to the time of day the flight was flown. Flights conducted in the hours immediately following dawn (7 a.m.–9 p.m. (GMT+8)) and preceding dusk (3 p.m.–5 p.m. (GMT+8)) appeared to produce the highest quality images.

## 4. Discussion

This study centered around the use of drone technology to complete an estuarine crocodile nesting survey of a section of the Kinabatangan River in Borneo. Positive identification was possible of five nests out of a total 29 potential locations; all other potential nests, not affected by flooding, were visually discounted on the ground. The approach, albeit in need of refinement, is a major improvement on the traditional techniques; for example, costly helicopter surveys entailing flying over large tracts of unsuitable habitat would still require post-event ground validation [4].

The identification of five nests within a relatively small (6710 ha) area of the study site suggests that crocodile females are actively selecting areas identified by the habitat suitability model as potential sites (Hypothesis 1). The use of such a model, as well as the selection of areas with open or semi-open canopy coverage, allowed for both highly selective and highly predictive flight mission planning (Hypothesis 3). The walked recces provided important justification of the use of drones in identifying potential nesting sites and crocodilian habitat (Hypothesis 2), and although the distance walked was relatively short (120 km), the transects’ placement to encompass tributaries, ox-bow lakes and other areas of permanent water sources (essential to successful nesting), provided a clear indicator that dense forest canopy does not represent important nesting habitat (Hypothesis 4). The identification of nests in close proximity to oil palm plantations demonstrates that females can nest in medium to high levels of human disturbance (Hypothesis 5).

While the identification of five nests provided validation of the methodology, the limited 35 km river stretch probably represented too small an area to provide a clear picture of the nesting habits of the crocodiles found throughout the LKWS. Nesting appears to be occurring at low densities, and whilst it is, at least to some extent, predictable, the presence of degraded, patchy secondary forest represented a challenge to successful and encompassing nesting surveys. As a result of breaks in forest canopy coverage, secondary forest results in far larger expanses of open areas than would likely to be present were the region to have retained its original primary forest landscape. As a result, refining search grids is more challenging in secondary forest ecosystems than in pristine primary forest habitats. Despite this reduction in canopy coverage, it is unlikely that all nests will be built in open areas and some crocodilians can use alternate heat sources such as termite mounds to keep their nests at the optimal temperatures [26]; however, to date, this has not been reported in *C. porosus*. The effect of such potential behaviours on the number of nests detected should, however, be negligible.

Flooding, as also determined by Webb et al. [3], is the primary threat to *C. porosus* nests in the Kinabatangan. Ten potential nests were completely submerged during the period of ground-verification, the river having risen in excess of 1 m over one night. There is evidence that increasing global temperatures associated with global warming could lead to a doubling of the frequency of El Niño events [27]. Unstable weather during nesting periods could lead to variability in successes and failures of nesting seasons.

The application of a GLMM was intended to inform what a model habitat would be for crocodile nesting in the LKWS. The model predictions provided less stringent buffers around major water sources than used in the original habitat suitability model and, as a result, the original cut off of 150 m (based on literature) used during the original “fuzzy overlay” model remained the best predictor of nesting habitat presence or absence. That only five nests were positively confirmed provided a too limited framework to generate statistically rigorous data for generic habitat features. Similarities between nest site choices and their spatial separation determined using the GLMM, and from observations, suggest that LKWS crocodiles are choosing sites preferentially and making active selections for nest locations (Hypotheses 1 and 3). That the nests of the 2013 survey were not reused, despite being successful, suggests either that females are not nesting annually or are not nest site-fidelic. There also appeared to be a general preference for smaller open areas, rather than large expanses of open grassland or swamp.

In carrying out this study, numerous UAV-related challenges were faced. While repeatability of transect observations is one of the major benefits of the technology over traditional techniques, the huge disparity in the resolution of the images produced in the two survey years is of concern. This variation could have resulted in the omission of potential nest sites during the 2014 surveys. Conversely, low-resolution images meant that a much larger number of “potential” nest sites needed to be ground-verified, as they could not be excluded due to poor image quality. A similar image resolution to that which was achieved during the 2013 surveys (of 5–6 cm per pixel) would have allowed for the exclusion of a number of the 2014 ‘potential’ nests. In addition, the number of nests located during this study provided limited statistical power. Expansion of the study range in order to provide a larger data set would allow for more wide-reaching conclusions to be made.

The crocodile population of the LKWS has endured fluctuations in both extent and stability; the current population size has, however, raised human-conflict concerns, with at least six anecdotal fatalities having occurred within the study area since 2010. The mapping of nesting habitats has a role in the mediation of conflict zones, especially if a further reduction in forest habitat results in a closer nesting proximity to human settlement. The identification of nests on an annual basis can also aid in the mapping of population trends. This, coupled with spotlighting surveys, could give a better indication of how the population is adapting to anthropogenic expansion. Nesting surveys of this nature could also provide estimates of the carrying capacity of both the study site and the LKWS as a whole, and how crocodile numbers could alter as forest conversion continues. An increase in sample size provided by annual nesting surveys would allow not only for a more in-depth modelling of nesting areas but also a more stringent predictive modelling. In this way, areas deemed most important to successful nesting could be protected, an action, which, in turn, would provide mediation of human-conflict issues. The status of crocodile populations throughout Sabah is also unclear so long term monitoring of nesting habits could be used as an indicator of ongoing population health.

Whilst in terms of cost, drone technology is far cheaper than many traditional survey methodologies, the main barrier to its use by small independent research projects is the cost of image stitching (a cost of around GBP1 per hectare (total area surveyed 6710 ha)). The number of images produced during flights prohibited the use of freeware image stitching software. These image data can be collected and utilised without the use of image stitching but detailed analysis of each specific image would require a far longer time-period. Additionally, the placing of any potential nest’s location within the broader context of the landscape, and assessing the hydrological relations, would be far more challenging and would require a highly specified knowledge of the study region.

In summary, the nests identified were spatially exclusive, showing that *C. porosus* individuals in the LKWS are not aggregate nesters. There was, however, an element of statistical predictability to nesting site locations in terms of distance from water bodies, thus allowing search area refinement. Nests were located at least several hundred meters from each other, and from any previously-used nesting sites. Nesting sites were found at sites of medium disturbance levels, although the presence of nest sites close to oil palm plantations suggests that human disturbance is not necessarily a barrier to nesting. This does suggest that stable estuarine crocodile populations could endure even in areas of moderate to high land-use conversion. In order to monitor the stability of the population in such areas, a long term monitoring programme is required.

## Figures and Tables

**Table 1:** “Fixed” and “random” model terms included in the binomial General Linear Mixed Model (GLMM) used to identify the most important factors in the presence or absence of crocodile nests. A logit link function was used for the model.

Dependent Variable	Fixed Model Terms	Random Model Terms
Presence of Nest (1/0)	Ground solidity	Year of detection
	Distance to water	
	Distance to canopy cover	
	Distance to plantation	
	Ground water presence	
